# Association between malnutrition, depression, anxiety and fatigue after stroke in older adults: a cross-lagged panel analysis

**DOI:** 10.1007/s40520-024-02892-7

**Published:** 2024-12-24

**Authors:** Hongmei Huang, Mengxia Lu, Pan Zhang, Lulu Xiao, Wanqiu Zhang, Yingjie Xu, Jinghui Zhong, Yiran Dong, Xian Chao, Yirong Fang, Jinjing Wang, Shiyi Jiang, Wusheng Zhu, Xinfeng Liu, Wen Sun

**Affiliations:** 1 Department of Neurology, Centre for Leading Medicine and Advanced Technologies of IHM, The First Affiliated Hospital of USTC, Division of Life Sciences and Medicine, University of Science and Technology of China, Hefei, Anhui 230001 China; 2 Department of Neurology, Nanjing Jinling Hospital, Nanjing Medical University, Nanjing, Jiangsu 210002 China; 3 Department of Neurology, Cixi People’s Hospital, Cixi, Zhejiang 315300 China; 4 Department of Neurology, Nanjing Jinling Hospital, Affiliated Hospital of Medical School, Nanjing University, Nanjing, Jiangsu 210002 China

**Keywords:** Malnutrition, Post-stroke depression, Post-stroke anxiety, Post-stroke fatigue, Cross-lagged panel model

## Abstract

**Background:**

Malnutrition, post-stroke depression (PSD), post-stroke anxiety (PSA), and post-stroke fatigue (PSF) in stroke survivors have complex relationships and are associated with adverse stroke outcomes.

**Aims:**

This research aims to explore the temporal and directional relationships between malnutrition, PSD, PSA, and PSF after stroke in older adults.

**Methods:**

Patients aged 65 years and older with their first ischemic stroke from two centers were selected and assessed at baseline, 3 months and 12 months. Malnutrition was evaluated using the Controlling Nutritional Status (CONUT) score, the Geriatric Nutritional Risk Index (GNRI), and the Prognostic Nutritional Index (PNI). PSD, PSA and PSF were measured with 24-item Hamilton Depression Scale (HAMD-24), 14-item Hamilton Anxiety Scale (HAMA-14) and Fatigue Severity Scale (FSS), respectively. The cross-lagged panel model (CLPM) was employed to investigate the temporal and directional relationships among these variables.

**Results:**

Among the 381 older patients included, 54.33%, 43.57%, and 7.87% were found to have malnutrition according to the CONUT, GNRI, and PNI scores, respectively. Significant bidirectional relationships were found between malnutrition and PSD, as well as between PSD, PSA, and PSF, but no significant bidirectional relationships between malnutrition, PSA and PSF were observed, irrespective of the malnutrition index used (CONUT, GNRI, or PNI).

**Conclusions:**

Nutritional status and post-stroke neuropsychiatric disorders in older stroke survivors are worthy of attention. Specifically, early malnutrition after stroke can predict later PSD and vice versa. PSD, PSA, and PSF are mutually predictable. Further studies are required to investigate the mechanisms of these findings.

**Supplementary Information:**

The online version contains supplementary material available at 10.1007/s40520-024-02892-7.

## Introduction

Stroke is the second leading cause of death globally. Although mortality has decreased due to the advancements of medical care, the long-term prognosis remains poor [1]. Almost all stroke survivors are affected by physical disabilities and neuropsychiatric disorders causing significant socioeconomic burdens [2].

The common neuropsychiatric disorders among stroke survivors are depression, anxiety and fatigue, occurring in at least 30% of cases [3]. Post-stroke depression (PSD) is the most prevalent, with an estimated prevalence ranging from 11 to 41% [4]. Post-stroke anxiety (PSA) is primarily characterized by symptoms of anxiety following a stroke, affecting at least one-third of post-stroke patients. Clinically, it manifests as excessive tension, worry, and fear after a stroke, with or without symptoms of autonomic nervous system hyperactivity [5]. Post-stroke fatigue (PSF) is characterized by a persistent lack of physical or mental energy that disrupts daily activities, with an estimated prevalence ranging from 25 to 85% [6]. Some research indicates a significant correlation between PSD and PSA [7–9]. Patients with PSA are more likely to develop PSD than those without anxiety [8]. Concurrently, studies have identified PSD and PSA as predictive factors for PSF [10–13]. This suggests a complex reciprocal relationship between PSD, PSA, and PSF. However, current research predominantly explores the unidirectional relationships between PSD, PSA, and PSF. The reciprocal influence among them warrants further investigation.

Malnutrition is common in ischemic stroke survivors and is significantly associated with higher risks of long-term mortality and severe disability [14, 15]. Research has found that malnutrition is linked to a higher prevalence of post-stroke neuropsychiatric disorders, although the precise mechanisms remain unclear [16, 17]. Furthermore, certain studies have identified post-stroke neuropsychiatric disorders as risk factors for malnutrition after stroke [18]. Therefore, the relationship between post-stroke neuropsychiatric disorders and malnutrition appears to be bidirectional [19]. Patients experiencing post-stroke neuropsychiatric disorders are more susceptible to malnutrition, and conversely, those with malnutrition face a heightened risk of developing post-stroke neuropsychiatric disorders. However, the temporal and directional relationships between malnutrition, PSD, PSA, and PSF have yet to be reported.

Considering the interactions between malnutrition, PSD, PSA, and PSF, this study aims to uncover the temporal and directional relationships between these variables in older adults after stroke at baseline, 3 months and 12 months, using cross-lagged panel analysis.

## Material and methods

### Study population

This two-center longitudinal observational cohort study. Patients with acute ischemic stroke (AIS) diagnosed within 14 days of the stroke event were consecutively recruited from February 2021 to February 2023.

Inclusion criteria for patients were (a) a diagnosis of their first-ever AIS within 14 days of onset, and (b) age 65 years or older. Exclusion criteria included (a) a history of psychiatric disorders, (b) severe dementia, aphasia, or loss of consciousness preventing the completion of neuropsychological tests, (c) presence of pre-stroke fatigue (defined as fatigue lasting 3 months or more before the index stroke), (d) known high-fatigue conditions such as hypothyroidism, multiple sclerosis, Parkinson’s disease, or anemia, (e) severe infections, severe liver or kidney dysfunction, tumors, or other conditions, (f) experiencing another stroke during the follow-up period.

### Baseline data collection

Baseline information on demographic characteristics, medical history, clinical features and stroke subtype was gathered by experienced neurologists upon admission. Body mass index (BMI) was determined by dividing weight in kilograms by height in meters squared (kg/m^2^). Medical history included hypertension, diabetes, smoking and alcohol drinking. Smoking was defined as having smoked at least 100 cigarettes by the date of inclusion and currently smoking or having quit smoking for less than 12 months. Drinking was defined as currently drinking alcohol or having previously consumed alcohol but having quit drinking for less than 12 months. The assessment and diagnosis of dysphagia are determined through clinical evaluation of swallowing function by experienced clinicians, with the option of utilizing additional instrumental diagnostics such as fiberoptic endoscopic evaluation of swallowing if required. Stroke subtypes were categorized according to the TOAST (Trial of ORG 10172 in Acute Stroke Treatment) criteria. Stroke severity was evaluated with the National Institutes of Health Stroke Scale (NIHSS). Additionally, the use of antidepressants was documented.

### Measures

Malnutrition, PSD, PSA, PSF were evaluated at baseline (T1), 3 months (T2) and 12 months (T3) via in-person or telephone interviews with patients and their caregivers by trained study staff. In instances where patients could not present at the research hospital, procurement of essential data for malnutrition assessment could be facilitated through the analysis of blood samples taken at proximate healthcare institutions.

#### Malnutrition screening tools

The Controlling Nutritional Status (CONUT) score includes measurements of serum albumin, total cholesterol, and lymphocyte count. Scores of 0–1 indicate no risk of malnutrition, 2–4 indicate mild risk, 5–8 indicate moderate risk, and 9–12 indicate severe risk [20].

The Geriatric Nutritional Risk Index (GNRI) is determined using the formula: 1.489 × serum albumin (g/L) + 41.7 × (current weight in kilograms / ideal weight). Ideal weight is calculated using Lorenz formulas: height (cm)－100－[(height (cm)－150)/4] for men and height (cm)－100－[(height (cm)－150)/2.5] for women. If weight exceeds ideal weight, set weight in kilograms / ideal weight to 1. Scores greater than or equal to 100 indicate no risk of malnutrition, 97.50–99.99 indicate a mild risk, 83.50–97.49 indicate a moderate risk, and less than 83.50 indicate a severe risk of malnutrition [21].

The Prognostic Nutritional Index (PNI) is derived from the formula: 10 × serum albumin (g/dl) + 0.005 × total lymphocyte count (mm^3^). Scores greater than 38 indicate no risk of malnutrition, 35–38 indicate moderate risk, and less than 35 indicate severe risk [22].

Patients were assessed for malnutrition using the above three indexes, respectively. Details of three indexes were presented in Table S1 and Table S2.

#### Post-stroke depression

PSD was evaluated using the 24-item Hamilton Depression Scale (HAMD-24)[23]. The HAMD-24, which includes 24 items with scores ranging from 0 to 72, has been validated as a reliable tool for assessing post-stroke depression. A higher score on the HAMD-24 indicates more severe symptoms of depression.

#### Post-stroke anxiety

PSA was evaluated using the 14-item Hamilton Anxiety Scale (HAMA-14) [24]. The HAMA-14 includes 14 items with a total score range of 0 to 56, confirmed and validated in previous studies. A higher score on the HAMA-14 indicates more severe symptoms of anxiety.

#### Post-stroke fatigue

PSF was evaluated using the Fatigue Severity Scale (FSS) [25]. The FSS contains 9 items, rated on a 1–7 scale from 'strongly disagree' to 'strongly agree', with total scores from 0 to 63. It has been confirmed as reliable for the Asian population. A higher score on the FSS indicates more severe symptoms of fatigue.

### Statistical analysis

Patient characteristics were summarized as mean ± SD or median (interquartile range) for continuous variables and as n (%) for categorical variables. Categorical variables were compared using the χ^2^ test or Fisher’s exact test. Continuous variables were analyzed using Student’s t-test or Mann–Whitney U test.

Spearman’s correlation analysis was utilized to investigate the correlations between malnutrition, PSD, PSA and PSF at three time points.

Chord diagrams were utilized to display the distribution of malnutrition across different BMI groups (underweight/normal weight and overweight/obesity) and temporal evolution of the assessed variables were also graphed.

To examine the temporal and directional relationships between malnutrition, PSD, PSA, and PSF, we employed cross-lagged panel model (CLPM). This approach allows for the investigation of reciprocal relationships and causal effects over time [26]. The CLPM, conducted using structural equation modeling, included covariates sex, age, hypertension, diabetes, smoking, drinking, TOAST, NIHSS scores, dysphagia and the use of antidepressants. Maximum Likelihood estimation with robust standard errors was employed to fit the models. The model fit was assessed using several fit indexes: (a) chi-square test of model fit / degrees of freedom (χ^2^/df) < 5, (b) comparative fit index (CFI) > 0.90, (c) Tucker-Lewis index (TLI) > 0.90, (d) standardized root mean square residual (SRMR) < 0.08, and (e) root mean square error of approximation (RMSEA) < 0.08 [27].

All data were analyzed by R 4.3.1 and Mplus 8.3, with two-sided significance set at P < 0.05.

## Results

### Baseline characteristics

This analysis included 381 patients over the age of 65 (Fig. S1). The majority of 381 patients were men (74.80%), with a mean age of 71.07 ± 4.27 years and median initial NIHSS of 3.00 (2.00–6.00). More detailed baseline characteristics of the participants are provided in Table 1.

**Table 1:** Baseline characteristics of study participants

Variables	Overall (n = 381)
Demographics	
Age, y	71.07 ± 4.27
Male	285 (74.80)
Height, cm	169.00 (163.00–173.00)
Weight, kg	70.00 (64.00–78.00)
BMI, kg/m^2^	24.80 (22.86–26.93)
Medical history	
Hypertension	268 (70.34)
Diabetes	151 (39.63)
Smoking	200 (52.49)
Drinking	146 (38.32)
Clinical features	
Dysphagia	116 (30.45)
Baseline NIHSS score	3.00 (2.00–6.00)
Stroke subtype (TOAST)	
LAA	159 (41.73)
SAA	126 (33.07)
CE	43 (11.29)
Other	53 (13.91)
Antidepressants	85 (22.31)
Laboratory feature	
Albumin, g/l	39.70 (37.60–42.40)
Total cholesterol, mg/dl	156.23 (129.54–187.94)
Lymphocyte, × 10^9^/l	1.68 (1.33–2.18)
Neuropsychological data	
FSS	24.00 (15.00–39.00)
HAMD-24	4.00 (2.00–9.00)
HAMA-14	3.00 (2.00–7.00)
Nutritional screening scores	
CONUT	2.00 (1.00–3.00)
GNRI	100.66 (97.24–104.24)
PNI	48.90 (45.45–52.60)

*BMI* Body mass index, *NIHSS* National Institutes of Health Stroke Scale, *TOAST* Trial of ORG 10172 in Acute Stroke Treatment, *CE* cardio-embolism, *LAA* large-artery atherosclerosis, *SAA* small-vessel occlusion, *FSS* Fatigue Severity Scale, *HAMD-24* 24-item Hamilton Depression Scale, *HAMA-14* 14-item Hamilton Anxiety Scale, *CONUT* controlling nutritional status score, *PNI* prognostic nutritional index, *GNRI* geriatric nutritional risk index

### Prevalence of malnutrition

Malnutrition prevalence among patients ranged from 7.87% according to the PNI, to 43.57% with the GNRI, and 54.33% with the CONUT (Table 2). The highest prevalence of malnutrition was found in patients categorized as underweight or normal weight based on BMI, with CONUT at 59.90%, GNRI at 65.66%, and PNI at 63.33% (Fig. S2). Notably, a significant proportion of patients classified as overweight or obesity also exhibited malnutrition, as indicated by CONUT (40.10%), GNRI (34.34%), and PNI scores (36.67%) respectively (Fig. S2).

**Table 2:** Prevalence of malnutrition at admission

	Malnutrition at Admission, n (%)			
	No	Mild	Moderate	Severe
CONUT	174 (45.67)	182 (47.77)	23 (6.04)	2 (0.52)
GNRI	215 (56.43)	66 (17.32)	93 (24.41)	7 (1.84)
PNI	351 (92.13)	–	21 (5.51)	9 (2.36)

*CONUT* controlling nutritional status score, *GNRI* geriatric nutritional risk index, *PNI* prognostic nutritional index

### Correlations between malnutrition, PSD, PSA, and PSF

**Fig. 1:**
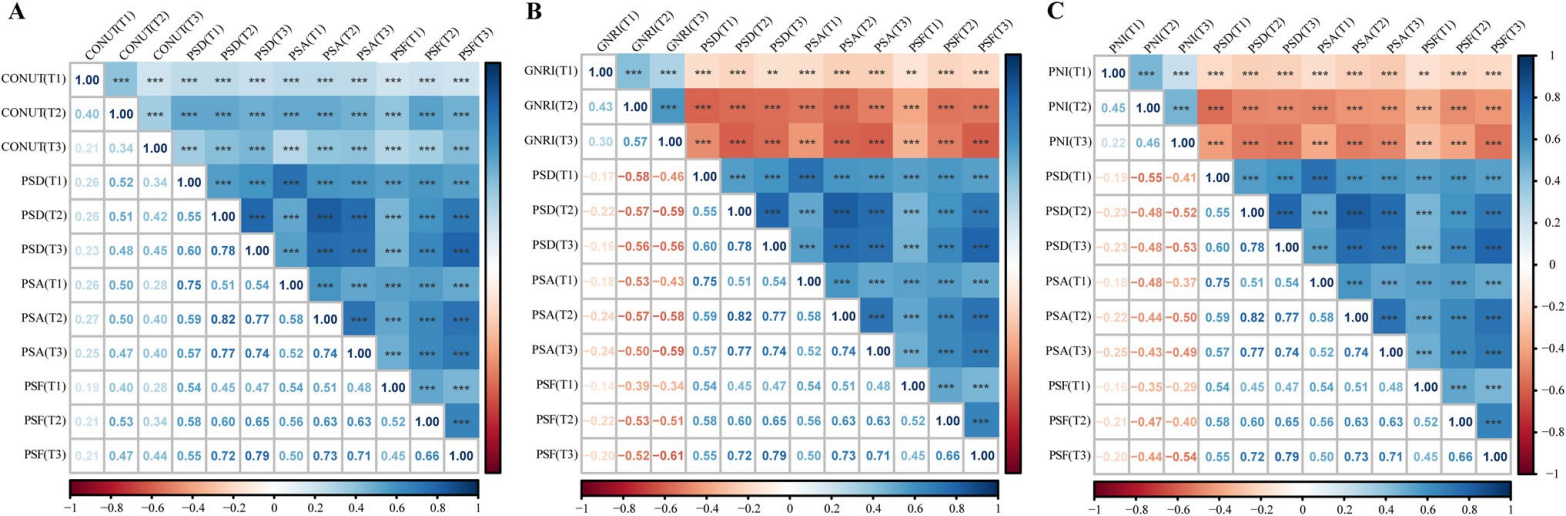
Correlations between Malnutrition (CONUT, GNRI, PNI), PSD, PSA and PSF. **A** Correlations using CONUT as malnutrition screening tool; **B** Correlations using GNRI as malnutrition screening tool; **C** Correlations using PNI as malnutrition screening tool. *CONUT* controlling nutritional status score, *PNI* prognostic nutritional index, *GNRI* geriatric nutritional risk index, *PSD* post-stroke depression, *PSA* post-stroke anxiety, *PSF* post-stroke fatigue. T1 = baseline; T2 = 3 months; T3 = 12 months. *** p < 0.001, ** p < 0.01

The changes of malnutrition, PSD, PSA and PSF at each time point are displayed in Fig. S3. The correlations between malnutrition (CONUT, GNRI and PNI), PSD, PSA, and PSF were presented in Fig. 1. The results demonstrated that malnutrition, PSD, PSA, and PSF were significantly interrelated at each time point, irrespective of the malnutrition screening tool utilized.

### The cross-lagged panel analysis

**Fig. 2:**
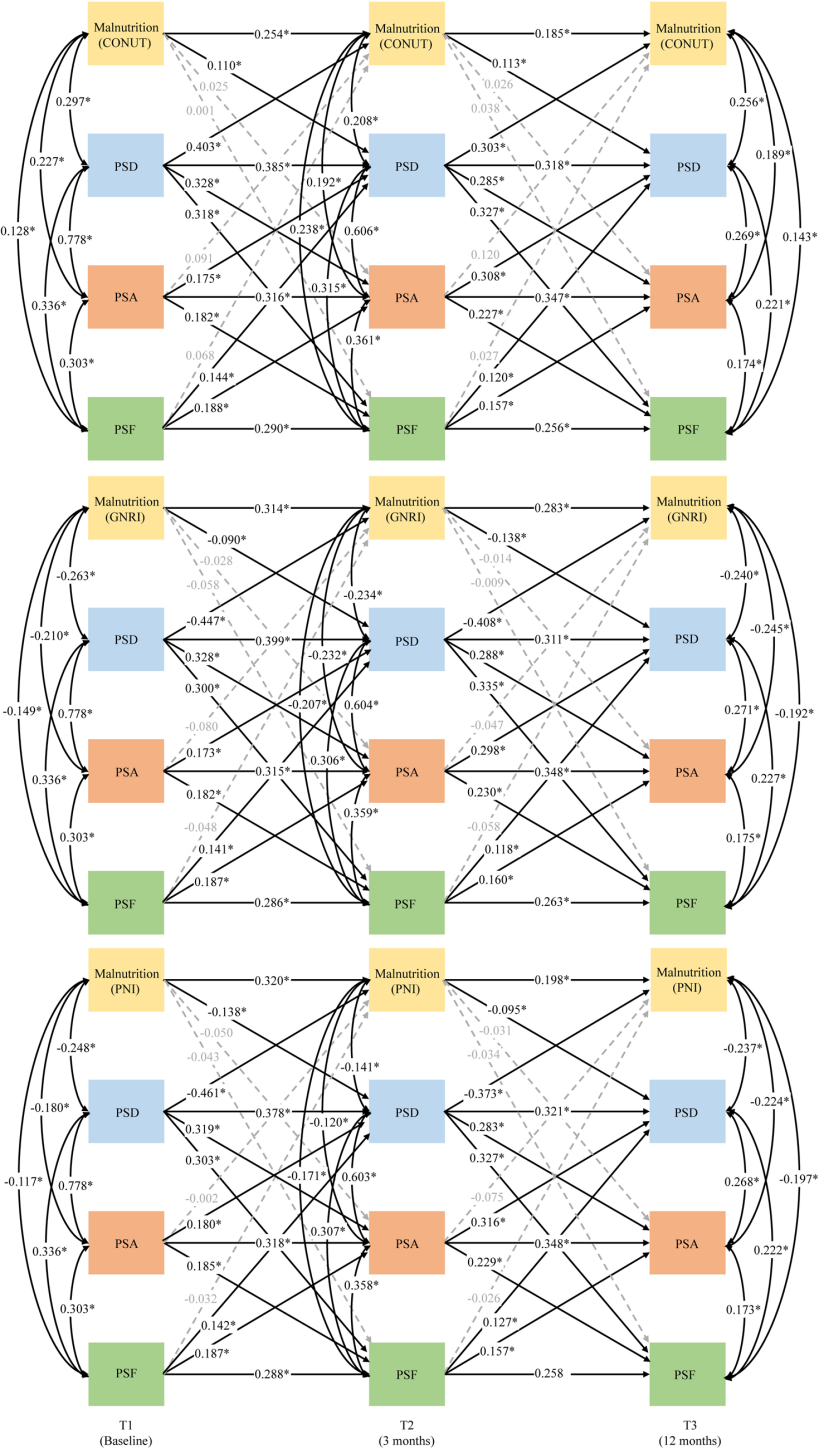
CLPM models between Malnutrition (CONUT, GNRI, PNI), PSD, PSA and PSF. *CONUT* controlling nutritional status score, *PNI* prognostic nutritional index, *GNRI* geriatric nutritional risk index, *PSD* post-stroke depression, *PSA* post-stroke anxiety, *PSF*, post-stroke fatigue. Adjusted for: sex, age, hypertension, diabetes, smoking, drinking, TOAST, NIHSS and the use of antidepressants. *p < 0.05

As illustrated in Fig. 2 and Table S3, significant autoregressive effects were observed from T1 to T2 and T2 to T3 for malnutrition (CONUT, GNRI, PNI), PSD, PSA, and PSF, with path coefficients ranging from 0.185 to 0.399 after controlling covariates. Moreover, significant correlational effects were observed between malnutrition (CONUT, GNRI, PNI), PSD, PSA, and PSF at T1, T2, and T3.

Regarding cross-lagged effects, after controlling the autoregression, as well as the correlation of four variables, significant bidirectional relationships were observed between PSD, PSA, and PSF from both T1 to T2 (*β*s = 0.141–0.328, *P*s < 0.05) and T2 to T3 (*β*s = 0.118–0.335, *P*s < 0.05) in three CLPM models. Moreover, significant bidirectional relationships were observed between malnutrition and PSD using different malnutrition screening tools (Fig. 2 and Table S3). When using CONUT, malnutrition was found to positively predict PSD from T1 to T2 (*β* = 0.110, *p* = 0.034) and from T2 to T3 (*β* = 0.113, *p* = 0.017); conversely, PSD significantly predicted malnutrition for both intervals (*β* = 0.403, *p* < 0.001 from T1 to T2; *β* = 0.303, *p* < 0.001 from T2 to T3). When using GNRI, where a lower score indicates greater risk of malnutrition, a significant inverse relationship was observed: a decrease in GNRI (indicating worse nutritional status) was associated with an increase in PSD from T1 to T2 (*β* = − 0.090, *p* = 0.036) and T2 to T3 (*β* = −0.138, *p* = 0.001); similarly, PSD was inversely predictive of malnutrition, indicating that higher levels of depression were linked to lower GNRI scores from both T1 to T2 (*β* = − 0.447, *p* < 0.001) and T2 to T3 (*β* = − 0.408, *p* < 0.001). When employing PNI reveal that malnutrition significantly predicted PSD from both T1 to T2 (*β* = − 0.138, *p* = 0.003) and T2 to T3 (*β* = − 0.095, *p* = 0.006), with PSD similarly predicting malnutrition from both T1 to T2 (*β* = − 0.461, *p* < 0.001) and T2 to T3 (*β* = − 0.373, *p* < 0.001).

Regardless of the malnutrition screening tools used, cross-lagged effects linking malnutrition to PSA or PSF were not found to be significant. Likewise, PSA and PSF were not significant predictors of malnutrition at subsequent time points (Fig. 2 and Table S3).

The fit indices for the three CLPM models were excellent. More details of model fitting are shown in Table S4.

## Discussion

This study revealed the following findings: (1) malnutrition is prevalent among stroke patients, as determined by three objective malnutrition scores; (2) a bidirectional relationship was observed between malnutrition and PSD in older adults. However, no significant bidirectional relationship was found between malnutrition and PSA, nor between malnutrition and PSF; (3) bidirectional relationships were observed between PSD, PSA, and PSF.

In recent years, the Controlling Nutritional Status (CONUT) score, the Geriatric Nutritional Risk Index (GNRI), and the Prognostic Nutritional Index (PNI) have been utilized for assessing patients with heart disease [28, 29]. The above three malnutrition screening tools synthesize the laboratory indexes such as serum albumin, total cholesterol, lymphocyte count, as well as height and weight parameters [20–22]. They possess good objectivity and accessibility, and are straightforward and useful tools for clinicians to identify individuals at risk of malnutrition. In this study, the prevalence of malnutrition varied from 7.87% to 54.33% among elderly stroke patients, consistent with previous studies reporting a prevalence ranging from 6.1% to 62% [30]. The variability in the prevalence of malnutrition risk can be ascribed to the distinct variables incorporated in each scoring system. The GNRI score integrates anthropometric measures, such as body weight, and serum markers, including albumin. The CONUT score, on the other hand, considers serum albumin, total cholesterol, and lymphocyte count, which reflect immune function. Conversely, the PNI score, although similar to CONUT, does not include cholesterol levels, which may explain the lower prevalence identified by PNI. Additionally, the PNI's exclusion of mild malnutrition in its assessment range might reduce its sensitivity to diagnosing malnutrition. Currently, clinicians follow guidelines that use lipid-lowering therapies to prevent cerebrovascular diseases. As a result, the inclusion of cholesterol levels in the CONUT score may lead to an overestimation of malnutrition risk prevalence. In our research, the prevalence assessed by CONUT was 54.33%, significantly higher than the 7.87% determined by PNI. Based on literature and our findings, we suggest that the GNRI score might be a more appropriate and objective tool for screening malnutrition risk in older AIS patients.

In this study, we also found that a significant proportion of patients classified as overweight or obesity were at risk of malnutrition. Typically, malnutrition is perceived as a condition resulting from undernutrition, leading to the misconception that it only affects underweight individuals. However, those with obesity can also experience malnutrition due to the loss of body composition, such as skeletal muscle, among other factors. In fact, being overweight or obese is acknowledged to be linked to nutritional deficiencies, although the underlying mechanisms are not fully understood [31, 32]. Recognizing this is crucial for the effective identification of diagnostic criteria.

Moreover, we found significant bidirectional relationships were observed between PSD and malnutrition over time, which means that more severe malnutrition was predictive of worsening PSD, and vice versa. This result is consistent with previous findings: early malnutrition after stroke predicts later PSD. Gu et al. verified that malnutrition was linked to a higher probability of incident PSD and was more likely to result in a slower decline in PSD risk [17]. Nutrients, including tryptophan, histidine, tyrosine, phenylalanine, and glutamic acid, choline, folic acid, vitamin B_12_, vitamin B_6_, play essential roles in the neuroendocrine functions and are necessary for the production of neurotransmitters involved in mood regulation, and deficiencies in them may contribute to the development of PSD [19, 33]. Furthermore, early PSD predicted later malnutrition, as stroke survivors with depression tend to have reduced energy intake along with other contributing factors.

Our study verified that there were no significant bidirectional relationships between malnutrition, PSA and PSF over time. A possible explanation is that anxiety is often closely related to psychological stress responses, environmental factors, and individual psychological states; meanwhile, fatigue may more often result from neuronal damage, muscle function decline, or changes in the central nervous system in stroke patients, which have weaker direct associations with nutritional status. Additionally, there is currently insufficient clinical evidence to determine whether malnutrition can predict anxiety and fatigue after stroke, and vice versa. A study on malnutrition and PSF found a negative correlation between PNI and FSS scores, with lower PNI increasing the risk of PSF [34]. However, that study employed a single malnutrition screening tool, assessing malnutrition solely at admission. In our study, we assessed malnutrition at three points in time and accounted for autoregressive effects using the CLPM model. The repeated measurements and longitudinal data in our study offer more robust evidence regarding the potential causal relationships between variables.

Additionally, our study uncovered significant bidirectional relationships among PSD, PSA, and PSF. Post-stroke neuropsychiatric disorders exhibit complex interrelations, frequently coexisting and impacting each other, linked to increased risks of recurrent stroke, disability, mortality, and reduced quality of life. Yet, they commonly remain overlooked and untreated [35]. We proved it and demonstrated that PSD, PSA and PSF are predictable and cause and effect of each other. This serves as a reminder for clinicians to pay closer attention to post-stroke neuropsychiatric disorders. Several explanations are possible for this finding. Research indicates that post-stroke depression, anxiety, and fatigue might stem from similar neurobiological disruptions, such as neurotransmitter imbalances that regulate mood, energy, and sleep [36–38]. Additionally, the stroke recovery process involves continuous physiological stress and inflammatory responses, contributing to these conditions [39–41]. Furthermore, stroke survivors often experience physical and quality of life declines, increasing depression and anxiety risks. Psychological stress and reduced coping capacity can intensify fatigue. These factors highlight the complex interplay between biological disruptions and psychosocial stressors in post-stroke emotional states and the specific mechanism needs further study.

Our study has a few advantages. Firstly, Primarily, as far as we know, this was the inaugural study to explore the temporal and directional relationships among malnutrition, depression, anxiety, and fatigue after stroke in older adults. Secondly, it was a two-center investigation and has a considerable sample size. Thirdly, our utilization of repeated measurements and longitudinal data, assessing malnutrition, PSD, PSA, and PSF at three distinct time points, enabled a detailed exploration of the changes and complex interrelationships among them.

There are several limitations to this study. Firstly, we only respectively chose a scale to assess PSD, PSA and PSF, so more scale will have better be combined and applied comprehensively in the future. Secondly, patients suffering from severe dementia, aphasia, or loss of consciousness preventing the completion of neuropsychological tests were excluded, limiting the applicability of our findings to all stroke patients. Thirdly, although CLPM can indicate potential causal relationships, establishing causality definitively necessitates corroborative studies using randomized controlled trials or other rigorous causal inference methodologies.

## Conclusion

In conclusion, this study identified significant bidirectional relationships between malnutrition and PSD, as well as among PSD, PSA, and PSF. However, no significant bidirectional relationships were found between malnutrition, PSA, and PSF. These findings underscore the importance for clinicians to closely monitor the nutritional status and address post-stroke neuropsychiatric disorders in stroke survivors. Future research is essential to elucidate the underlying mechanisms of these relationships.

## Supplementary Information

Below is the link to the electronic supplementary material.

Supplementary file1 (DOCX 630 KB)

## Data Availability

The datasets used and/or analyzed during the current study are available from the corresponding author upon reasonable request.
