# Supplementary material for: Association between malnutrition, depression, anxiety and fatigue after stroke in older adults: a cross-lagged panel analysis
Source: Aging Clin Exp Res. 2024 Dec 24;37(1):4. doi: 10.1007/s40520-024-02892-7 (PMC11668841; doi:10.1007/s40520-024-02892-7)
Supplement: Supplementary file 1 — Supplementary file1 (DOCX 630 KB) [file 40520_2024_2892_MOESM1_ESM.docx]

**Supplementary File**

**Association between malnutrition, depression, anxiety and fatigue after stroke** **in older adults: A cross-lagged panel analysis**

Hongmei Huang, et al.

**Correspondence to**

Xinfeng Liu

Department of Neurology, Centre for Leading Medicine and Advanced Technologies of IHM, The First Affiliated Hospital of USTC, Division of Life Sciences and Medicine, University of Science and Technology of China, Hefei, Anhui, 230001, China

Telephone number: +86 13813835114

E-Mail: xfliu2@ustc.edu.cn

Wen Sun

Department of Neurology, Centre for Leading Medicine and Advanced Technologies of IHM, The First Affiliated Hospital of USTC, Division of Life Sciences and Medicine, University of Science and Technology of China, Hefei, Anhui, 230001, China

Telephone number: +86 15050589620

E-Mail: sunwen_medneuro@163.com

**Table S1.** Formula of malnutrition screening tools.

| **Screening tools** | | **Formula** | | | | |
| --- | --- | --- | --- | --- | --- | --- |
| **CONUT, points** | | |  |  |  |  |
| Serum albumin (g/l) | | | ≥35 | 30-34.9 | 25-29.9 | <25 |
| Score | | | 0 | 2 | 4 | 6 |
| Total cholesterol (mg/dl) | | | ≥180 | 140-179 | 100-139 | <100 |
| Score | | | 0 | 1 | 2 | 3 |
| Lymphocyte (×10^9^/l) | | | ≥1.60 | 1.20-1.59 | 0.80-1.19 | <0.80 |
| Score | | | 0 | 1 | 2 | 3 |
| **GNRI, points** | 1.489 × serum albumin(g/l) + 41.7 × (weight in kilograms/ideal weight) ^a^ | | | | | |
| **PNI, points** | 10 × serum albumin (g/dl) + 0.005 × total lymphocyte count (mm^3^) | | | | | |

Abbreviations: CONUT, controlling nutritional status score; PNI, prognostic nutritional index; GNRI, geriatric nutritional risk index.

^a^ Ideal weight is calculated using Lorenz formulas: height (cm) - 100 - [(height (cm) - 150)/4] for men and height (cm) - 100 - [(height (cm) - 150)/2.5] for women. If weight exceeds ideal weight, set weight in kilograms / ideal weight to 1.

**Table S2.** Risk of malnutrition according three screening tools.

|  | Risk of Malnutrition | | | |
| --- | --- | --- | --- | --- |
|  | No | Mild | Moderate | Severe |
| CONUT, points | 0-1 | 2-4 | 5-8 | 9-12 |
| GNRI, points | ≥100 | 97.50-99.99 | 83.50-97.49 | <83.50 |
| PNI, points | >38 | - | 35-38 | <35 |

Abbreviations: CONUT, controlling nutritional status score; PNI, prognostic nutritional index; GNRI, geriatric nutritional risk index.

**Table S3.** Estimates from three CLPM between malnutrition indexes (CONUT, NRI, PNI), PSD, PSA and PSF.

|  |  | M=CONUT | | |  | M=GNRI | |  | M=PNI | |
| --- | --- | --- | --- | --- | --- | --- | --- | --- | --- | --- |
|  |  | *β* | | *p* |  | *β* | *p* |  | *β* | *p* |
| Cross-lagged effects | |  |  | |  |  |  |  |  |  |
| T1 M 🡪 T2 PSD | | 0.110 | **0.034** | |  | -0.090 | **0.036** |  | -0.138 | **0.003** |
| T1 M 🡪 T2 PSA | | 0.025 | 0.557 | |  | -0.028 | 0.441 |  | -0.050 | 0.163 |
| T1 M 🡪 T2 PSF | | 0.001 | 0.980 | |  | -0.058 | 0.176 |  | -0.043 | 0.358 |
| T1 PSD 🡪 T2 M | | 0.403 | **<0.001** | |  | -0.447 | **<0.001** |  | -0.461 | **<0.001** |
| T1 PSD 🡪 T2 PSA | | 0.328 | **0.001** | |  | 0.328 | **0.001** |  | 0.319 | **0.001** |
| T1 PSD 🡪 T2 PSF | | 0.318 | **0.001** | |  | 0.300 | **0.001** |  | 0.303 | **0.001** |
| T1 PSA 🡪 T2 M | | 0.091 | 0.375 | |  | -0.080 | 0.307 |  | -0.002 | 0.979 |
| T1 PSA 🡪 T2 PSD | | 0.175 | **0.037** | |  | 0.173 | **0.044** |  | 0.180 | **0.033** |
| T1 PSA 🡪 T2 PSF | | 0.182 | **0.032** | |  | 0.182 | **0.032** |  | 0.185 | **0.032** |
| T1 PSF 🡪 T2 M | | 0.068 | 0.152 | |  | -0.048 | 0.234 |  | -0.032 | 0.471 |
| T1 PSF 🡪 T2 PSD | | 0.144 | **0.001** | |  | 0.141 | **0.001** |  | 0.142 | **0.001** |
| T1 PSF 🡪 T2 PSA | | 0.188 | **<0.001** | |  | 0.187 | **<0.001** |  | 0.187 | **<0.001** |
| T2 M 🡪 T3 PSD | | 0.113 | **0.017** | |  | -0.138 | **0.001** |  | -0.095 | **0.006** |
| T2 M 🡪 T3 PSA | | 0.026 | 0.607 | |  | -0.014 | 0.761 |  | -0.031 | 0.377 |
| T2 M 🡪 T3 PSF | | 0.038 | 0.462 | |  | -0.009 | 0.847 |  | -0.034 | 0.463 |
| T2 PSD 🡪 T3 M | | 0.303 | **<0.001** | |  | -0.408 | **<0.001** |  | -0.373 | **<0.001** |
| T2 PSD 🡪 T3 PSA | | 0.285 | **0.004** | |  | 0.288 | **0.004** |  | 0.283 | **0.005** |
| T2 PSD 🡪 T3 PSF | | 0.327 | **<0.001** | |  | 0.335 | **<0.001** |  | 0.327 | **<0.001** |
| T2 PSA 🡪 T3 M | | 0.120 | 0.134 | |  | -0.047 | 0.516 |  | -0.075 | 0.307 |
| T2 PSA 🡪 T3 PSD | | 0.308 | **0.002** | |  | 0.298 | **0.003** |  | 0.316 | **0.002** |
| T2 PSA 🡪 T3 PSF | | 0.227 | **0.001** | |  | 0.230 | **0.001** |  | 0.229 | **0.001** |
| T2 PSF 🡪 T3 M | | 0.027 | 0.645 | |  | -0.058 | 0.259 |  | -0.026 | 0.647 |
| T2 PSF 🡪 T3 PSD | | 0.120 | **0.006** | |  | 0.118 | **0.012** |  | 0.127 | **0.006** |
| T2 PSF 🡪 T3 PSA | | 0.157 | **0.001** | |  | 0.160 | **0.002** |  | 0.157 | **0.001** |
| Autoregressive effects | |  |  | |  |  |  |  |  |  |
| T1 M 🡪 T2 M | | 0.254 | **<0.001** | |  | 0.314 | **<0.001** |  | 0.320 | **<0.001** |
| T1 PSD 🡪 T2 PSD | | 0.385 | **<0.001** | |  | 0.399 | **<0.001** |  | 0.378 | **<0.001** |
| T1 PSA 🡪 T2 PSA | | 0.316 | **0.001** | |  | 0.315 | **0.001** |  | 0.318 | **0.001** |
| T1 PSF 🡪 T2 PSF | | 0.290 | **<0.001** | |  | 0.286 | **<0.001** |  | 0.288 | **<0.001** |
| T2 M 🡪 T3 M | | 0.185 | **0.001** | |  | 0.283 | **<0.001** |  | 0.198 | **<0.001** |
| T2 PSD 🡪 T3 PSD | | 0.318 | **0.003** | |  | 0.311 | **0.004** |  | 0.321 | **0.003** |
| T2 PSA 🡪 T3 PSA | | 0.347 | **0.002** | |  | 0.348 | **0.002** |  | 0.348 | **0.002** |
| T2 PSF 🡪 T3 PSF | | 0.256 | **<0.001** | |  | 0.263 | **<0.001** |  | 0.258 | **<0.001** |

Abbreviations: M, malnutrition; CONUT, controlling nutritional status score; PNI, prognostic nutritional index; GNRI, geriatric nutritional risk index; PSD, post-stroke depression; PSA, post-stroke anxiety; PSF, post-stroke fatigue. T1 = baseline; T2 = 3 months; T3 = 12 months.

**Table S4.** Fit indices of three CLPM models.

|  | χ^2^ | df | CFI | TLI | SRMR | RMSEA (90%CI) |
| --- | --- | --- | --- | --- | --- | --- |
| CONUT | 29.275 | 16 | 0.995 | 0.945 | 0.010 | 0.047(0.017-0.073) |
| GNRI | 28.809 | 16 | 0.996 | 0.951 | 0.010 | 0.046(0.016-0.072) |
| PNI | 22.543 | 16 | 0.998 | 0.973 | 0.009 | 0.033(0.000-0.062) |

Abbreviations: CONUT, controlling nutritional status score; PNI, prognostic nutritional index; GNRI, geriatric nutritional risk index; χ^2^, chi-square; df, degrees of freedom; TLI, Tucker-Lewis index; CFI, comparative fit index; SRMR, standardized root mean square residual; RMSEA, root mean square error of approximation.

**Figure S1.** Flow chart.


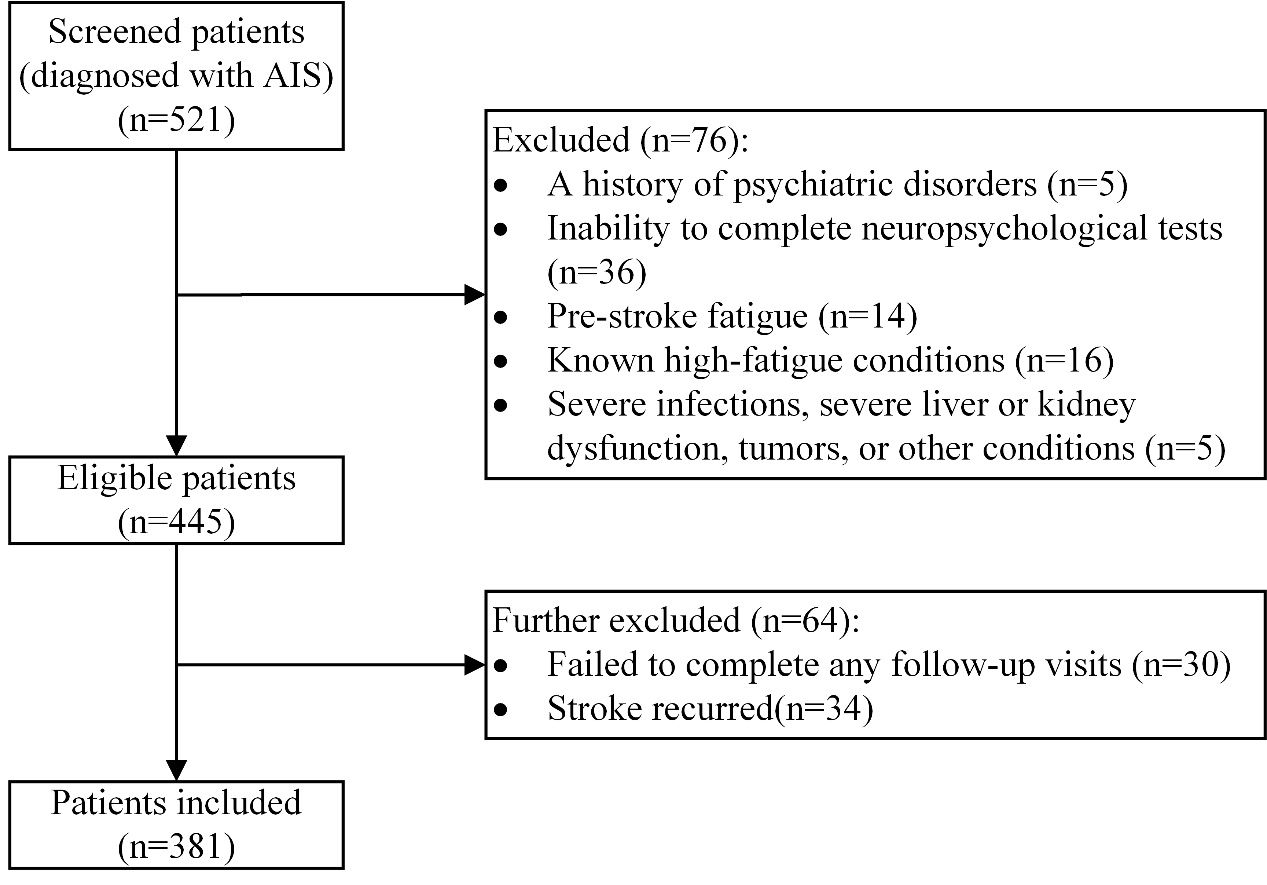


**Figure S2.** Prevalence of different degrees of malnutrition according to BMI.


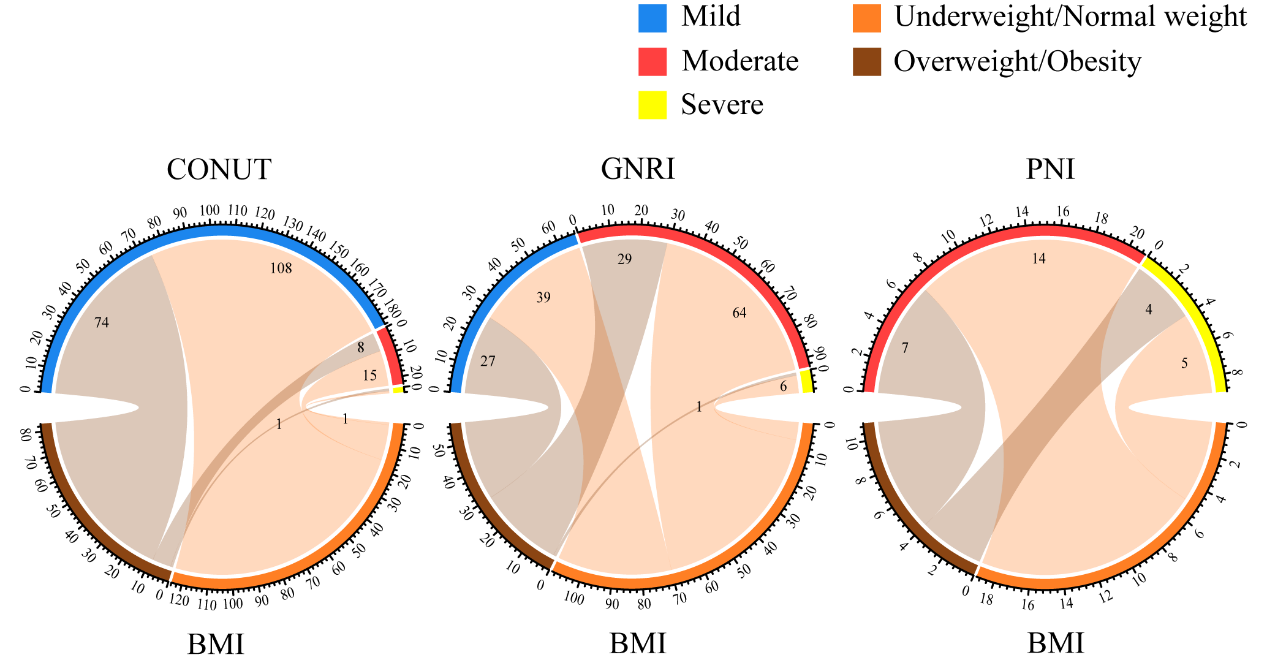


In the chord diagrams, the connections in pink represent patients with malnutrition who were classified as underweight/normal weight, whereas the connections in brown indicate patients who were classified as overweight/obesity. The numbers are absolute number of patients for each measure. Abbreviations: CONUT, controlling nutritional status score; GNRI, geriatric nutritional risk index; PNI, prognostic nutritional index; BMI, body mass index.

**Figure S3.** Changes in variables over time.


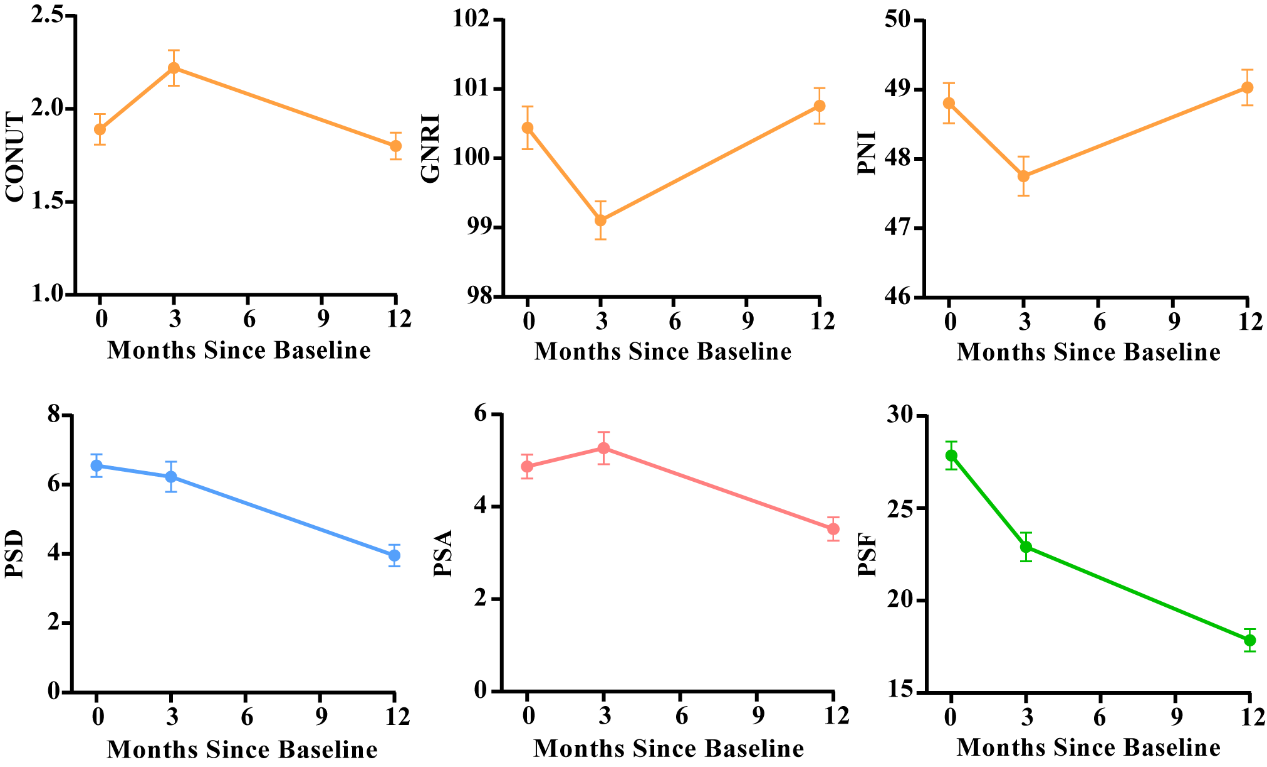


Each point on the line represents the mean value of the variable at a specified time point post-baseline, with error bars reflecting the standard errors of the mean, indicating the variability within the sample. Abbreviations: CONUT, controlling nutritional status score; GNRI, geriatric nutritional risk index; PNI, prognostic nutritional index; PSD, post-stroke depression; PSA, post-stroke anxiety; PSF, post-stroke fatigue.
